# Development of a nomogram to predict 5-year tooth loss after root canal treatment in patients with cracked teeth and chronic irreversible pulpitis

**DOI:** 10.2340/aos.v85.46624

**Published:** 2026-08-07

**Authors:** Qian Dong, Shiliang Guo

**Affiliations:** a Department of Specialist Clinic, Nanjing Stomatological Hospital, Affiliated Hospital of Medical School, Institute of Stomatology, Nanjing University, Nanjing, China; b Department of Endodontics, Nanjing Stomatological Hospital, Affiliated Hospital of Medical School, Institute of Stomatology, Nanjing University, Nanjing, China

**Keywords:** Cracked teeth, chronic irreversible pulpitis, root canal treatment, tooth retention, nomogram model

## Abstract

**Objective:**

To analyze the influencing factors of tooth retention within 5 years after root canal treatment (RCT) in patients with cracked teeth and chronic irreversible pulpitis, and to construct a nomogram model so as to provide a basis for the selection of clinical treatment regimen for such patients.

**Methods:**

This retrospective case analysis included 295 patients (295 affected teeth) with cracked teeth complicated with chronic irreversible pulpitis who were treated from January 2017 to January 2020. Tooth retention within 5 years was taken as outcome event; independent-samples *t* test and chi-square test were applied for single factor comparison. Lasso was used to screen the variables. Binary logistic regression analysis was utilized to analyze the related factors. The nomogram model was constructed by the ‘rms’ package of R software. Receiver operating characteristic (ROC) curve was drawn to evaluate the discrimination degree of the model. Hosmer-Lemeshow (H-L) test was performed and the calibration curve was drawn to evaluate the calibration degree of the model. Decision curve analysis was conducted to evaluate the clinical practicability of the model.

**Results:**

Successful and improved teeth were recorded as tooth survival. According to whether the teeth were retained within 5 years, the enrolled patients were categorized into survival group (265 cases) and non-survival group (30 cases), with the 5-year tooth survival rate of 89.9%. Multivariate analysis identified 2–3 RCT visits (Odds Ratio (OR) [95% Confidence Interval (CI)] = 0.15 [0.05, 0.50]), probing depth > 5 mm (OR [95% CI] = 2.99 [1.26, 7.09]), and crown restoration (OR [95% CI] = 0.28 [0.09, 0.83]) as independent predictors of 5-year tooth loss (*p* < 0.05). The nomogram was constructed based on multivariate results and the area under the ROC curve of the prediction model was 0.73 (0.62,0.84). H-L test revealed *p* = 0.682, and the calibration curve of the model had a high overlap degree with the ideal curve. The risk threshold ranged from 4 to 68%. The nomogram model line was located at the upper right of the None line and the All line.

**Conclusion:**

Multivariate analysis identified 2–3 RCT visits, probing depth > 5 mm, and crown restoration as independent predictors of 5-year tooth loss, and the nomogram constructed based on the above indicators has good practicability to predict tooth loss in patients. However, the sample size of our study was relatively limited, and the patient population was specific and may not be representative of the general population. Further external validation in a multi-center population is warranted.

## Introduction

Endodontic diseases are common and frequently-occurring diseases in oral clinics, and they are among the three major non-communicable diseases for prevention and treatment along with cardiovascular diseases and cancer [1]. Chronic irreversible pulpitis is the most common clinical condition, typically not causing severe spontaneous pain, but presenting with paroxysmal dull pain or daily regular dull pain. It has a prolonged course, with the affected teeth often accompanied by occlusal discomfort or mild percussion pain. When acute episode occurs, it is manifested as acute pulpitis symptoms [2]. Studies have suggested that nearly 80% of patients are treated due to acute toothache, with over half of these cases directly attributable to pulp and periapical diseases [3]. Dental pulp disease is a chronic and progressive disease causing severe pain in acute episodes. Without timely treatment to the affected teeth in the stage of dental pulp disease, the condition will develop into periapical disease. In severe cases, it will lead to tooth loss, affecting masticatory and digestive functions. If the condition affects jaws and adjacent tissues, it causes infection and lesions of other distant organs, thus affecting the overall body health.

Cracked tooth syndrome typically occurs in individuals aged 45–60 years, with a higher prevalence rate in mandibular molars. It has become an important cause of toothache and dentition loss. Patients with cracked teeth complicated with chronic irreversible pulpitis often experience more severe pain, seriously impacting their daily work and life [4]. Tooth cracks refer to the non-physiological cracks on the surface of the crown, which are not easily noticeable and irreparable. The concept of cracked teeth is similar to cracked teeth syndrome, which Cameron defines as an incomplete fracture of the posterior teeth, usually involving the dentin and sometimes extending into the pulp [5]. There is much disagreement regarding the most common tooth locations of cracked teeth, but most scholars believe that the most susceptible teeth are mandibular molars, maxillary premolars, maxillary molars, mandibular premolars and anterior teeth. The mandibular molars have the characteristics of early eruption, steep cusp, deep fissure, and high masticatory force. The continuous impact of the thick and palatal tip of the maxillary molars leads to fatigue of the mandibular molar structure, which is considered to be the main factor leading to tooth cracking.

Currently, root canal treatment (RCT) is the preferred method for the treatment of dental pulp disease and periapical disease in clinical practice [6–8]. RCT can effectively enhance the long-term retention rate of cracked teeth with chronic irreversible pulpitis, but there are still some patients with long-term tooth loss in clinical practice [9]. Currently, a large number of scholars have explored and studied the key factors affecting the efficacy of RCT, primarily including the root canal system anatomical factors, preoperative pulp and periapical states, periapical lesion size, root canal filling-related factors, and postoperative crown restoration quality [10]. However, there are few long-term follow-up studies on the evaluation of RCT efficacy and its influencing factors, especially comprehensive big data analysis. As a predictive assessment tool, the nomogram model is helpful to screen high-risk factors and identify high-risk groups. It is a statistical model that quantifies the risk probability of multiple predictors and events [11]. The development of a nomogram model helps medical staff not only to predict the probability of occurrence of a health condition but also to objectively and effectively identify the main factors causing the condition. This ultimately helps them formulate appropriate and personalized intervention measures [12, 13]. The purpose of this study is to explore the factors influencing tooth retention after RCT in patients with cracked teeth and chronic irreversible pulpitis, and to construct a nomogram model to more intuitively predict the risk of tooth loss in patients and provide reference for clinical decision-making.

## Methods

### Study design and patients

This retrospective case analysis included 295 patients (295 affected teeth) with cracked teeth complicated with chronic irreversible pulpitis who were treated from January 2017 to January 2020. All the included affected teeth met the indications of RCT. Regarding sample size, we assessed the adequacy of our sample using the events-per-variable (EPV) criterion. With 30 outcome events and 4 candidate predictors selected by Lasso, the EPV was 7.5, which is below the commonly recommended threshold of 10–20. However, we employed Lasso regression for variable selection and used bootstrap-corrected performance estimates to mitigate overfitting, which is consistent with the TRIPOD guideline’s recommendations for handling limited sample sizes.

Patients included in this study were those whose affected teeth were diagnosed as cracked teeth with clinically diagnosed chronic irreversible pulpitis. The clinical diagnosis of chronic irreversible pulpitis was based on the presence of the following symptoms and signs: (1) a history of prolonged, intermittent dull pain or discomfort, often with daily regularity; (2) mild to moderate percussion pain or occlusal discomfort; (3) absence of severe spontaneous pain typical of acute irreversible pulpitis; and (4) radiographic findings that may include deep caries or restoration approaching the pulp, with or without periapical radiolucency. Notably, the term ‘chronic irreversible pulpitis’ used in this study refers to a clinical diagnostic category corresponding to a chronic course of pulp inflammation, which is distinct from the histological definition. We acknowledge that the current international nomenclature more commonly classifies pulpitis as reversible or irreversible pulpitis based on clinical symptoms and pulp vitality status. In our cohort, all included patients presented with clinical features consistent with a chronic course of irreversible pulpitis, characterized by persistent dull pain and prolonged symptoms without the classic acute exacerbation pattern [14]. The clinical diagnostic criteria for cracked teeth were subjective symptoms unexplained by other causes, positive iodine tincture staining test, positive tooth transillumination test and positive bite test. Meeting any three of these criteria confirmed the diagnosis of cracked teeth. All the included patients were those who received RCT for the first time, and their ages ranged from 18 to 85 years.

**Figure 1:**
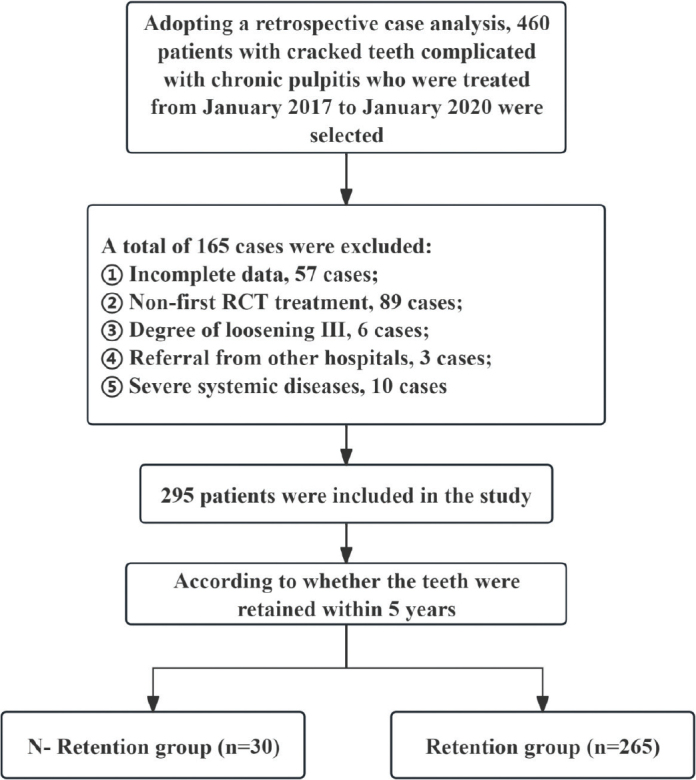
Flow chart of the process used in this study for patient record inclusion.

Patients were excluded if the affected teeth had undergone vital pulpotomy before enrollment and had tooth fracture and crack. This study excluded pregnant and lactating women and patients diagnosed with any mental illness or cognitive impairment. Referral of initial treatment in other hospitals was not permitted. Patients suffering from serious systemic diseases, such as severe atrioventricular block, hypertension, atherosclerosis, arrhythmia, hyperthyroidism, diabetes mellitus, and various types of heart disease, were also excluded. Patients with incomplete follow-up data were eliminated. At the same time, patients with loosening of degree III and those with insufficient filling quality were excluded. The flow chart of patient inclusion and exclusion criteria is shown in Figure 1.

### Treatment methods

#### Clinical data

The study subjects who met the inclusion criteria were identified through outpatient logs and electronic medical records. All predictor variables were extracted retrospectively from the electronic medical records by two trained research assistants independently, and discrepancies were resolved by consensus with a third senior clinician. To ensure data quality, a standardized data extraction form was developed prior to data collection, detailing the definition and categorization of each variable. The clinical data included age, gender, crown restoration (yes/no; defined as a full-coverage crown placed within 6 months after RCT completion), number of RCTs (categorized as 1, 2–3, or ≥ 4 visits), pulp floor crack (yes/no, determined by intraoperative microscopic examination), periapical status (normal vs. periapical radiolucency, assessed on preoperative periapical radiographs), probing depth (> 5 mm vs. ≤ 5 mm, measured at six sites per tooth using a periodontal probe), tooth position (premolar vs. molar), location (maxilla vs. mandible), tooth mobility (none, I degree, II degree), crack distribution (classified according to the involved marginal ridges and grooves), root canal length (mm), root canal sealing material, root canal filling material, root canal filling quality (fit and proper vs. overfill), ultrasonic irrigation (yes/no), treatment cycle (days from pulp opening to root canal filling), preoperative Visual Analogue Scale (VAS) [15], postoperative 12 hours VAS, and postoperative 24 hours VAS. The treatment cycle is defined as the number of days from opening the pulp to root canal filling treatment.

#### Treatment process

All RCT procedures were performed by three experienced endodontic specialists, each with more than 10 years of clinical experience in endodontics. Prior to the start of this study, all operators participated in a calibration session to standardize the treatment protocol, including access cavity preparation, root canal instrumentation, irrigation protocols, and obturation techniques, based on the departmental standard operating procedures. Inter-operator consistency was assessed through a pilot test of 10 cases, and the agreement on key procedural steps exceeded 90%. Throughout the study period, treatment decisions were made based on individual patient conditions at the discretion of the treating specialist, following the same clinical guidelines. Preoperative periapical radiographs were taken for all affected teeth receiving RCT before treatment. All affected teeth were treated with routine removal of necrotic tissue, opening pulp, uncovering pulp, cleaning and repairing pulp cavity, and establishing a straight access. Firstly, the root canal orifice was preliminarily explored with root canal probe DG16 or hand stainless steel K file, and 10#/15# hand stainless steel K file was used to dredge and clean the root canal. After that, the initial apical file was determined and combined with root canal length measuring instrument to determine the working length or the initial apical file periapical radiograph was taken to determine the working length. Thereafter, root canal preparation, root canal irrigation and disinfection, root canal filling and crown restoration were carried out. According to the different conditions of the affected teeth and personal preference of the surgeons, different root canal preparation instruments and ethylenediaminetetraacetic acid (EDTA) were selected for root canal preparation. During root canal preparation, 1 mL of 3% sodium hypochlorite at 60°C was used for root canal irrigation every time the instrument was replaced. After the completion of root canal preparation, each group was irrigated with 5 mL of normal saline for 1 minute, followed by 1 mL of 17% EDTA as the final irrigation for 1 minute. Finally, the root canal was irrigated with 5 mL of distilled water, and was dried with paper point. Ultrasonic irrigation was performed with ultrasonic working tip 15# to stir 3% sodium hypochlorite and irrigate for 30 seconds. The whole RCT process was completed once, twice or multiple times according to the conditions of the affected teeth. For affected teeth with one-visit RCT, root canal filling was implemented after moisture-separated drying. For affected teeth with multiple-visit RCT, root canal sealing was performed to temporarily seal the cavities after moisture-separated drying. The temporary sealing materials and root canal sealing drugs were removed during return visit, and moisture-separated drying and root canal filling were carried out after the corresponding treatment. The root canal sealer used in this study was injectable with bioceramic technology. Periapical radiographs were taken after root canal filling. Crown restoration was performed within 6 months after the RCT.

#### Return visit data

Return visit data included clinical symptoms, signs and imaging findings of the affected teeth. The clinical and imaging follow-ups of the affected teeth were carried out at 3 months, 6 months, 12 months, 2 years, 3 years, 4 years and 5 years after the treatment completion of the affected teeth. The follow-up deadline was January 2025, and the follow-up time was recorded from the completion of RCT to the latest return visit. The follow-up contents were consisted of the following three aspects: subjective symptoms of the affected teeth, clinical signs of the affected teeth and imaging findings of the affected teeth. The return visit was conducted by the attending physician and the contents of the return visit were recorded in detail. The subjective symptoms were obtained from the medical records or oral statements of patients, and the clinical symptoms and signs were derived from the medical records or clinical examinations of the attending physician.

#### Efficacy evaluation

The primary outcome was tooth survival within 5 years after RCT completion, defined as the affected tooth remaining in situ and functionally retained without extraction. Outcome assessment was performed at each follow-up visit (3 months, 6 months, 12 months, 2 years, 3 years, 4 years, and 5 years post-treatment) by the attending physician based on a combination of subjective symptoms, clinical examination, and periapical radiographs. The outcome classification was independently reviewed by two endodontic specialists who were blinded to the predictor variables. Disagreements were resolved through discussion. The follow-up deadline was January 2025. The treatment was considered successful if patients had no subjective symptoms, good masticatory function, good root canal filling on periapical radiographs, and no abnormalities in periapical, periodontal and root images. Improvement of the affected teeth was indicated if patients had mild discomfort without spontaneous pain, acceptable masticatory function, and no significant changes in the periodontal and periapical areas on periapical radiographs. Treatment failure of the affected teeth was considered if patients had one or more of the following symptoms or signs: obvious subjective symptoms of spontaneous pain, swelling and bite pain, new areas of bone destruction around the apex of the affected teeth, unhealed or expanded original apical shadow within 5 years on periapical radiographs, significant root canal filling defects requiring crown restoration, and tooth fracture and crack. Affected teeth classified as successful or improved were recorded as effectively retained.

### Principles of scientific research ethics

This study was approved by the ethics committee of Nanjing Stomatological Hospital, Affiliated Hospital of Medical School, Institute of Stomatology, Nanjing University (Approval No: NJSH-2024NL-046) and was conducted in accordance with the principles of the Declaration of Helsinki. This study adhered to the principles of confidentiality and anonymity and responsible data handling.

### Statistical analysis

SPSS 27.0 and Graphpad Prism 9.5 were utilized to analyze data. Enumeration data were expressed as (*n* [%]). The Chi-square test was used to determine the association between categorical variables if the expected cell frequencies (counts) were ≥ 5, otherwise the Fisher’s exact test was used. The Shapiro-Wilk test was used to assess whether the data were normally distributed or not, with data conforming to normality summarized by mean ± SD, and data consistent with non-normal distribution were denoted as Median (M) (lower quartile [Q1], upper quartile [Q3]), and *t* test and Mann-Whitney *U* test were performed for comparison respectively. Lasso regression was first employed for variable selection using the ‘glmnet’ package in R software. The tuning parameter λ was selected as lambda.min via 10-fold cross-validation. Variables with nonzero coefficients at the optimal λ were retained as candidate predictors. Subsequently, a binary logistic regression model was fitted using these candidate predictors. Variables with a *p*-value < 0.05 in the multivariate logistic regression were retained in the final prediction model. A nomogram was then constructed based on the final logistic regression model using the ‘rms’ package in R software. The nomogram assigned points to each predictor variable according to its regression coefficient, and the total points were used to estimate the predicted probability of tooth loss within 5 years. Receiver operating characteristic (ROC) curve was adopted to assess the discrimination degree of the model, and Hosmer-Lemeshow (H-L) goodness-of-fit test and calibration curve after 1000 times of Bootstrap method repeated sampling were applied to evaluate the calibration degree of the model, and decision curve was adopted to evaluate the clinical application value of the model. The cut-off value was selected according to the best Youden index. Test level was considered significant at α = 0.05. This prediction model study was reported in accordance with the TRIPOD checklist for prediction model development (without external validation). To address potential confounding by indication, whereby disease severity may influence both treatment decisions and outcomes, we employed the following strategies. First, Lasso regression was used for variable selection to mitigate overfitting and reduce the impact of multicollinearity among correlated predictors. Second, multivariate logistic regression was adjusted for clinically relevant variables including age, probing depth, and periapical status to account for differences in baseline disease severity. Third, variance inflation factor (VIF) was calculated to assess multicollinearity, with all VIF values below 1.1, indicating no serious collinearity among the included predictors. However, we acknowledge that residual confounding from unmeasured indicators of disease severity cannot be fully excluded due to the retrospective nature of this study.

## Results

### Clinical characteristics of cracked teeth with chronic irreversible pulpitis

Based on tooth retention status within 5 years, the included patients were classified into survival group (265 cases) and non-survival group (30 cases), with the 5-year tooth survival rate of 89.9%. The clinical characteristics of the two groups are shown in Table 1. Group (89.9%) exhibited significant differences in the number of RCTs (Fisher’s exact probability < 0.001), age (p = 0.023), crown restoration (Fisher’s exact probability = 0.003) and probing depth > 5 mm (p = 0.001). No significant differences were observed in other clinical characteristics (all p > 0.05).

**Table 1:** Clinical characteristics and difference analysis.

Variables	Total (*n* = 295)	Non-survival group (*n* = 30)	Survival group (*n* = 265)	*P*
Age, years, *M* (*Q*₁, *Q*₃)	57.00 (47.00, 65.00)	64.50 (54.25, 69.00)	57.00 (47.00, 65.00)	0.023
Root canal length, mm, *M* (*Q*₁, *Q*₃)	18.20 (16.70, 20.40)	18.60 (17.40, 20.15)	18.20 (16.60, 20.50)	0.720
Treatment cycle, *M* (*Q*₁, *Q*₃)	22.00 (20.00, 25.00)	22.00 (20.25, 25.75)	22.00 (20.00, 25.00)	0.803
Preoperative VAS, points, *M* (*Q*₁, *Q*₃)	3.00 (3.00, 3.00)	3.00 (3.00, 3.00)	3.00 (3.00, 3.00)	0.300
Postoperative 12 hours VAS, points, *M* (*Q*₁, *Q*₃)	3.00 (2.00, 3.00)	3.00 (2.00, 3.00)	3.00 (2.00, 3.00)	0.678
Postoperative 24 hours VAS, points, *M* (*Q*₁, *Q*₃)	2.00 (1.00, 2.00)	2.00 (1.00, 2.00)	2.00 (1.00, 2.00)	0.998
Gender, *n* (%)				0.739
Female	156 (52.88)	15 (50.00)	141 (53.21)	
Male	139 (47.12)	15 (50.00)	124 (46.79)	
Crown restoration, *n* (%)				0.003
No	27 (9.15)	8 (26.67)	19 (7.17)	
Yes	268 (90.85)	22 (73.33)	246 (92.83)	
Number of RCTs, *n* (%)				< 0.001
≥ 4	17 (5.76)	7 (23.33)	10 (3.77)	
1	14 (4.75)	3 (10.00)	11 (4.15)	
2~3	264 (89.49)	20 (66.67)	244 (92.08)	
Pulp floor crack, *n* (%)				0.171
No	268 (90.85)	25 (83.33)	243 (91.70)	
Yes	27 (9.15)	5 (16.67)	22 (8.30)	
Periapex, *n* (%)				0.359
No abnormality	69 (23.39)	5 (16.67)	64 (24.15)	
Shadow	226 (76.61)	25 (83.33)	201 (75.85)	
Probing depth > 5 mm, *n* (%)				0.001
No	234 (79.32)	17 (56.67)	217 (81.89)	
Yes	61 (20.68)	13 (43.33)	48 (18.11)	
Tooth position, *n* (%)				0.229
Dentes premolars	87 (29.49)	6 (20.00)	81 (30.57)	
Molar	208 (70.51)	24 (80.00)	184 (69.43)	
Location, *n* (%)				0.153
Upper jaw	210 (71.19)	18 (60.00)	192 (72.45)	
Lower jaw	85 (28.81)	12 (40.00)	73 (27.55)	
Tooth mobility, *n* (%)				0.424
No	242 (82.03)	23 (76.67)	219 (82.64)	
I degree	37 (12.54)	6 (20.00)	31 (11.70)	
II degree	16 (5.42)	1 (3.33)	15 (5.66)	
Crack distribution, *n* (%)				0.468
Crossing the mesiodistal marginal ridge	76 (25.76)	10 (33.33)	66 (24.91)	
Proximal marginal ridge	97 (32.88)	9 (30.00)	88 (33.21)	
Crossing the palatal sulcus	39 (13.22)	6 (20.00)	33 (12.45)	
Crossing the buccal groove	33 (11.19)	3 (10.00)	30 (11.32)	
Distal marginal ridge	50 (16.95)	2 (6.67)	48 (18.11)	
Filling quality				0.190
Fit and proper	287 (97.29)	28 (93.33)	259 (97.74)	
Over charge	8 (2.71)	2 (6.67)	6 (2.26)	
Root canal filling material, *n* (%)				
Other	15 (5.08)	1 (3.33)	14 (5.28)	1.000
Gutta-percha point	280 (94.92)	29 (96.67)	251 (94.72)	
Ultrasonic irrigation, *n* (%)				0.551
No	94 (31.86)	11 (36.67)	83 (31.32)	
Yes	201 (68.14)	19 (63.33)	182 (68.68)	

*M*: Median; *Q*₁: lower quartile; *Q*₃: upper quartile; RCT: root canal treatment.

### Results of multivariate analysis

**Figure 2:**
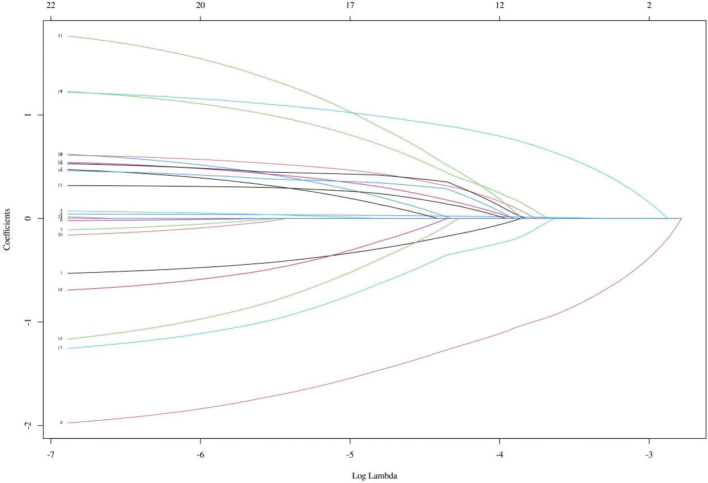
Lambda plot of the lasso regression.

**Figure 3:**
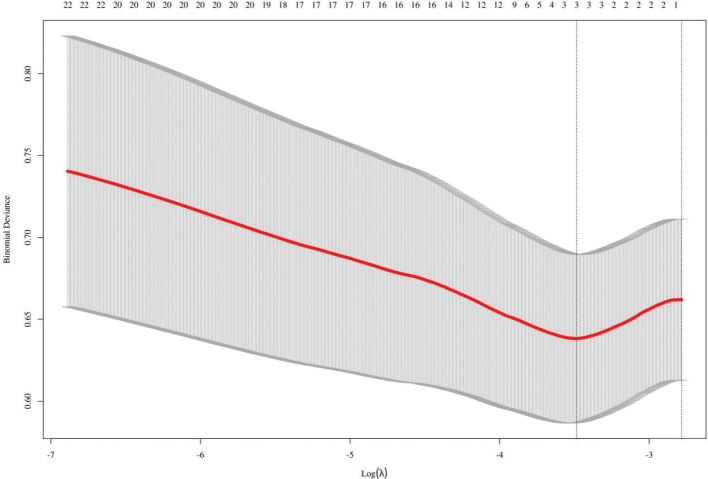
Cross-validation results from lasso regression.

Taking the tooth retention status within 5 years as the outcome event (survival = 0, non-survival = 1), lasso was used to screen the variables, input nlambda = 1000, and choose λ value as lambda.min. The lambda plot is shown in Figure 2, and the lasso fit cross-validation results are shown in Figure 3. Lasso regression identified four variables with nonzero coefficients at the optimal λ: age (beta = 0.003), crown restoration (beta = –0.662), number of RCTs (beta = 0.993), and probing depth (beta = 0.466). These four variables were then entered into a multivariate binary logistic regression analysis. As shown in Table 2, three variables remained statistically significant at *p* < 0.05: number of RCTs of 2–3 times (OR [95% CI] = 0.15 [0.05, 0.50], *p* = 0.002), probing depth > 5 mm (OR [95% CI] = 2.99 [1.26, 7.09], *p* = 0.013), and crown restoration (OR [95% CI] = 0.28 [0.09, 0.83], *p* = 0.021). Age was not statistically significant (*p* = 0.063) and was not retained in the final model. Therefore, the final prediction model included three predictors: number of RCTs, probing depth, and crown restoration.

**Table 2:** Multivariate logistic regression results.

Outcomes	Multivariate analysis				
	β	S.E	*Z*	*P*	OR (95% CI)
Intercept	–1.73	1.27	–1.37	0.171	0.18 (0.01, 2.11)
Age	0.03	0.02	1.86	0.063	1.03 (1.00, 1.07)
Number of RCTs					
≥ 4					1.00 (Reference)
1	–1.31	0.92	–1.43	0.154	0.27 (0.04, 1.63)
2~3	–1.87	0.60	–3.12	0.002	0.15 (0.05, 0.50)
Probing depth > 5 mm					
No					1.00 (Reference)
Yes	1.09	0.44	2.48	0.013	2.99 (1.26, 7.09)
Crown restoration					
No					1.00 (Reference)
Yes	–1.27	0.55	–2.31	0.021	0.28 (0.09, 0.83)

RCT: root canal treatment.

### Establishment of prediction model

**Figure 4:**
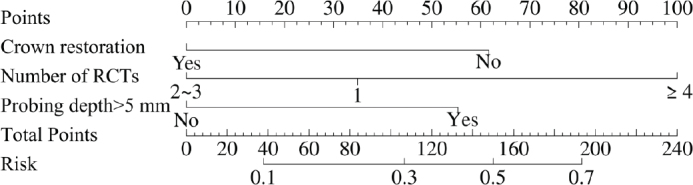
Prediction model of tooth loss within 5 years after RCT in patients with cracked teeth and chronic irreversible pulpitis. RCT: root canal treatment.

VIF test displayed that the VIF values of number of RCTs, probing depth, age and crown restoration were 1.014, 1.038, 1.055 and 1.026 respectively, suggesting the absence of multicollinearity. On this basis, the nomogram model was constructed through the ‘rms’ package of R software. From Figure 4, it could be seen that the total score of the nomogram ranged from 0 to 240 points, and the occurrence probability of tooth loss within 5 years after RCT in patients with cracked teeth and chronic irreversible pulpitis was between 0.1 and 0.7.

### Validation of prediction model

**Figure 5:**
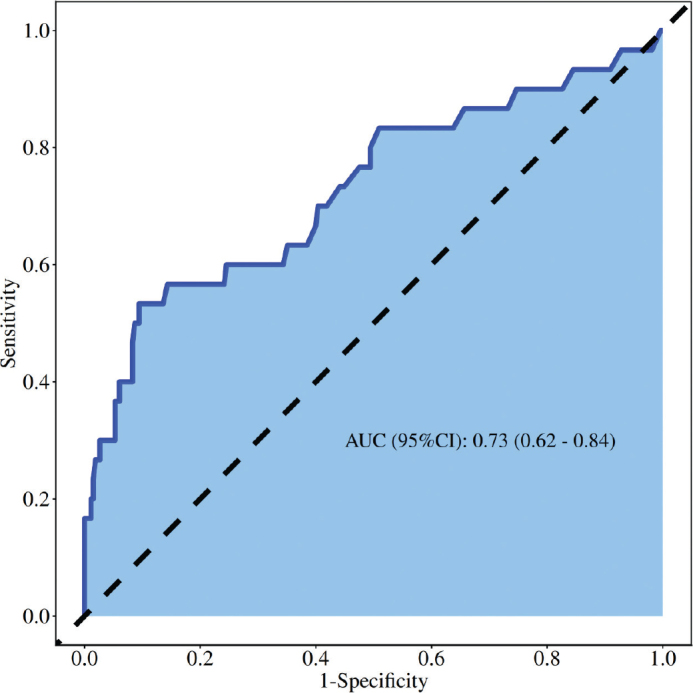
ROC curve. ROC: receiver operating characteristic.

**Figure 6:**
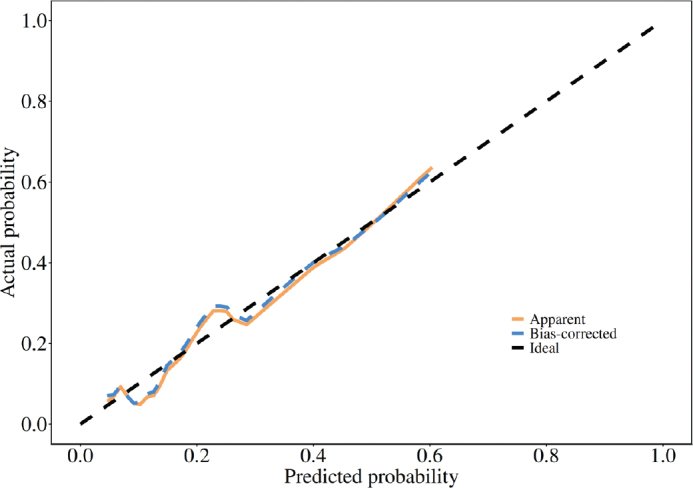
Calibration curve.

**Figure 7:**
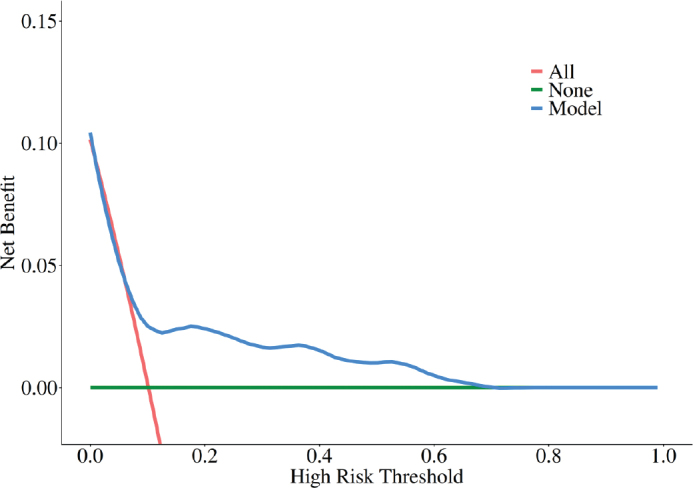
Decision curve.

As shown in Figure 5, the area under the ROC curve (AUC) of the prediction model was 0.73 (0.62, 0.84),the Accuracy (95% CI), Sensitivity (95% CI), Specificity (95% CI), F1 score, Brier score and cut-off were 0.87 (0.82, 0.90), 0.53 (0.35, 0.71), 0.91 (0.87, 0.94), 0.45 (0.08, 0.165). This indicates that the prediction model of tooth loss within 5 years after RCT in patients with cracked teeth and chronic irreversible pulpitis constructed in this study demonstrates a good discrimination degree. H-L test and calibration curve were combined to evaluate the calibration degree of the risk prediction nomogram model. H-L test results revealed that X-squared = 5.68, df = 8, and *p*-value = 0.682. The ideal line represented the ideal curve, the bias-corrected line represented the calibration curve obtained by bootstrap, and the apparent line represented the original curve of the nomogram model. The higher the overlap degree between the calibration curve and the ideal curve, the better the performance of the nomogram model. From Figure 6, it could be seen that the calibration curve of the model exhibited a high overlap degree with the ideal curve. The None line indicated that none of patients used the risk prediction model, resulting in a net benefit rate of 0. The All line revealed that all patients adopted the risk prediction model, and the net benefit was a negative backslash. The nomogram model line represented the net benefit of patients under the risk prediction model constructed in this study. As shown in Figure 7, when the risk threshold was between 4 and 68%, the nomogram model line was located at the upper right of the None line and the All line, and the model could provide significant additional clinical net benefits, indicating that the risk prediction model constructed in this study has good clinical practicability.

## Discussion

This study found that the tooth survival rate within 5 years after RCT was 89.9% in patients with cracked teeth and chronic irreversible pulpitis. The number of RCTs of twice or three times, probing depth > 5 mm and crown restoration were independently related to tooth loss within 5 years after RCT in patients with cracked teeth and chronic irreversible pulpitis. This study constructed a nomogram based on multivariate results, and revealed that AUC of the prediction model was 0.73 (0.62, 0.84). H-L test in this study showed that *p* = 0.682, and the calibration curve of the model had a high overlap degree with the ideal curve. The nomogram model line was located at the upper right of the None line and the All line when the risk threshold ranged between 4 and 68%. The nomogram constructed in this study has good discrimination degree and clinical practicability in predicting tooth loss.

This study did not involve microbial detection. The following is the literature review content. The common reasons for RCT failure include the following factors. (1) Microbial factors: *Enterococcus faecalis*, *Candida albicans*, etc. Additionally, open drainage procedure will cause the communication between the oral cavity and the root canal system, facilitating the entry of oral bacteria into the root canal, thus leading to secondary mixed infections with a more complex bacterial composition than endogenous root canal bacteria. (2) Anatomical factors: including curved root canal, abnormal root canal morphology and number. (3) Iatrogenic factors: root canal missing, poor quality of root canal filling, poor coronal sealing, and complications (instrument separation, root canal perforation, root canal deviation, root canal step, etc.) [16, 17]. At present, there are many studies on the efficacy of RCT, but the success rate of RCT is not the same due to the different efficacy evaluation contents, methods, standards and time used in different studies. It has been reported that the cumulative retention rates of the affected tooth at 10 years, 20 years, 30 years and 37 years after initial RCT are 97, 81, 76 and 68% respectively [18]. With the application of microscopic root canal therapy technique, dentists can utilize the illumination and amplification provided by microscope to more clearly observe the depth of the crack during the treatment of cracked teeth, observe whether the crack extends to the pulp floor, and record it in the medical record, so as to provide a basis for dentists to evaluate the prognosis of the affected tooth and make treatment decisions. The prognosis of cracked teeth depends on the depth of the crack. Sim et al. found that the 5-year tooth survival rate of cracked teeth with a perfect RCT was 95.2% when the crack only existed in the crown, and the tooth survival rate was only 81.8% when the crack extended to the pulp floor or deeper. The expansion of the crack involving the pulp chamber floor increased the probability of eventual tooth extraction by 11 times [19]. When a deep root crack occurs in the affected tooth or the depth of the crack involves the pulp chamber floor, clinicians should inform patients of the possibility of treatment failure and that the affected tooth may eventually need to be extracted. The success rate of RCT in the 295 patients (295 affected teeth) included in this study was 89.9%. The imaging evaluation method used in this study was periapical radiograph. Due to the limitations of its two-dimensional imaging, periapical radiograph cannot visualize buccal and lingual lesions and is prone to overlapping with adjacent tissues, leading to potential misinterpretation. Furthermore, studies have indicated that the presence of lesions can only be observed in periapical radiograph when bone loss reaches 30–50%, potentially leading to an overestimation of RCT success rate in this study.

RCT can be classified into one-visit RCT, two-visit RCT and multiple-visit RCT. Currently, in clinical practice, one-visit RCT is predominantly adopted for patients with infection involving only pulp tissue, while RCT is often carried out twice or even multiple times for patients with lesions involving periapical tissue. At present, there is a great controversy about one-visit, two-visit and multiple-visit methods of RCT. A study has considered that compared with multiple-visit method, one-visit method cannot completely eliminate the bacteria in the root canal system, and its efficacy for the treatment of affected teeth with periapical lesions before surgery is significantly lower than that of multiple-visit methods. Therefore, the root canal should be sealed and disinfected during treatment to further eradicate the bacteria in the root canal system, thereby enhancing the efficacy of RCT [20]. The findings in this study suggested that there was a significant difference in the number of RCTs between the non-survival group and the survival group, and the number of RCTs of twice or three times was an independent influencing factor for tooth retention, but no significant difference was found in the relationship between one-visit RCT and tooth retention. The reason for the consideration of number of RCTs is that the disease conditions of patients with cracked teeth and chronic irreversible pulpitis in this study are complex, and most patients have periapical lesions at the time of consultation. Due to the presence of cracked teeth and occlusal trauma of the affected teeth, the periapical lesions are not easy to heal and the bacteria in the root canal system are not easy to remove, so multiple-visit RCT is required to thoroughly eliminate the infectious substances in the root canal system [21, 22]. However, it is worth noting that when other conditions permit, reducing the number of RCTs in patients and shortening the time from the beginning of treatment to the completion of full crown restoration as much as possible can effectively improve the long-term prognosis of patients.

Previous studies have demonstrated that cracked teeth treated with RCT have a higher retention rate. However, the presence of periodontal pockets associated with cracks often leads to a higher risk of tooth loss [23]. Krell et al. report that periodontal probing depth greater than 5 mm was one of the main reasons for the failure of treatment for cracked teeth, with a success rate of only 74.1%, and the three most important factors in multivariate analysis were pocket depth, distal marginal ridge crack and periapical diagnosis [24]. The results of this study are consistent with the above-mentioned findings. The prognosis of cracked teeth is influenced by multiple factors, such as the location, direction, size or degree of cracks. Additionally, periodontal probing depth is also a significant clinical factor in determining the prognosis of patients with cracked teeth [25]. The primary purpose of the treatment of cracked teeth is to protect the hard tissues of the teeth and prevent further expansion of the lesions. Therefore, in the diagnosis and treatment of cracked teeth in clinical work, the location, depth, degree and range of cracked teeth should be carefully examined, the depth of periodontal pocket should be carefully explored, and the treatment indications and prognosis should be carefully evaluated. Crown restoration can effectively protect the cusp tissue, improve the fracture resistance of the tooth, further reduce or eliminate the masticatory stress, and avoid the problem of local bite force concentration, thus preventing further expansion of the tooth crack. The results of this study support the view that patients receiving crown restoration have better long-term prognosis. Therefore, for patients who do not receive crown restoration, doctors should strengthen the follow-up and review, urge patients to accept crown restoration as far as possible, and instruct them to avoid chewing hard food. It should be noted that this study adopted tooth survival as the primary outcome rather than strict endodontic success. This choice was made because cracked teeth inherently present a compromised prognosis due to structural defects, and even teeth with successful endodontic outcomes may exhibit persistent symptoms or functional limitations. Tooth survival, as a composite endpoint encompassing both asymptomatic and mildly symptomatic functioning teeth, better reflects real-world clinical outcomes and aligns with previous studies on cracked teeth. However, we acknowledge that this definition may overestimate the true success rate of RCT, as teeth categorized as ‘improved’ may still have suboptimal function.

In summary, this retrospective study identified the number of RCT visits, probing depth > 5 mm, and crown restoration as factors associated with 5-year tooth loss in patients with cracked teeth and chronic irreversible pulpitis. The nomogram constructed based on these three predictors showed moderate discriminative ability (AUC 0.73) and good calibration in this cohort. However, due to several important methodological limitations discussed below, the clinical utility of this model should be regarded as preliminary and exploratory at this stage. First, as a retrospective study, this design inherently carries methodological risks, including potential selection bias, information bias, and unmeasured confounding. The data were collected from electronic medical records, which may be subject to incomplete or inconsistent documentation. Although Lasso regression was employed to screen variables and reduce overfitting, residual confounding cannot be fully excluded. Prospective studies with standardized data collection protocols are warranted to validate our findings. Second, the sample size is relatively limited, and the patient population has its relative particularity and may not represent the general population. Further external validation is needed in a multi-center population. The difference in survey population may lead to differences between the findings of this study and those of other studies. Considering that a hyperglycemic environment can affect the treatment outcome of RCT in multiple ways, we excluded diabetic patients, which may limit its promotion to the elderly population with common systemic diseases. Future studies can further expand the sample size and include more patients from different regions and different age groups to verify and refine the accuracy and applicability of the prediction model. Another important methodological limitation is that the number of RCT visits and crown restoration were included as predictors in the prediction model, yet these factors represent treatment decisions rather than baseline patient characteristics. In clinical practice, patients with a severe disease are more likely to require multiple RCT visits and may have a lower likelihood of receiving crown restoration due to a poorer prognosis. This introduces the potential for confounding by indication. While we adjusted for available clinical variables in the multivariate analysis and assessed multicollinearity, we cannot fully exclude the possibility that the observed associations between these treatment-related factors and tooth loss are partially attributable to the underlying disease severity rather than the treatment itself. Future prospective studies with standardized treatment protocols stratified by disease severity are needed to disentangle the effects of treatment from those of baseline disease characteristics. With only 30 tooth loss events among 295 patients, the effective sample size for multivariate analysis was limited. Simulation studies have suggested that an EPV of at least 10 is generally required to obtain reliable estimates in logistic regression models. The EPV of 7.5 in this study falls below this threshold, which may have led to two potential issues. First, there is an elevated risk of Type I error, meaning that some of the significant predictors identified in our model may be chance findings. Second, the limited number of events increases the risk of Type II error, meaning that some clinically relevant predictors may have been overlooked due to insufficient statistical power. For example, age showed a borderline association (*p* = 0.063) in the multivariate analysis, and the one-visit RCT category (*p* = 0.154) had a relatively wide confidence interval, both of which may reflect insufficient power rather than a true lack of association. Therefore, our findings should be considered exploratory, and validation in larger cohorts is essential. Additionally, although standardized data extraction forms and independent dual-review were employed, the retrospective nature of data collection may still have introduced information bias and temporal bias, particularly regarding the timing of predictor measurement and outcome ascertainment. This study did not fully adhere to all 13 steps recommended by Efthimiou for clinical prediction model development. Specifically, the study protocol was not pre-registered; we did not conduct a formal sample size calculation a priori, and the findings have not been externally validated in an independent cohort [26]. These aspects should be addressed in future studies before the nomogram can be recommended for routine clinical application. At present, the model may serve as a hypothesis-generating tool rather than a validated clinical decision aid.

## References

[1] Zhang CF, Wang J, Zhu XY, Wang N. Effect of ultrasonic irrigation combined with epoxy resin paste in single visit root canal treatment in chronic pulpitis. J Coll Physicians Surg Pak. 2023;33(10):1130–5. 10.29271/jcpsp.2023.10.1130 37804018

[2] Shetty P, Bhat R, Padavu S, Rai P, Kumar BK, Shetty S. Molecular profiling of antibiotic resistance genes in acute and chronic irreversible pulpitis: a cross-sectional study. J Conserv Dent Endod. 2025;28(8):814–20. 10.4103/JCDE.JCDE_311_25 40860388 PMC12377663

[3] Ricucci D, Siqueira JF, Jr, Abdelsayed RA, Lio SG, Rôças IN. Bacterial invasion of pulp blood vessels in teeth with symptomatic irreversible pulpitis. J Endod. 2021;47(12):1854–64. 10.1016/j.joen.2021.09.010 34597722

[4] Kakka A, Gavriil D, Whitworth J. Treatment of cracked teeth: a comprehensive narrative review. Clin Exp Dent Res. 2022;8(5):1218–48. 10.1002/cre2.617 35809233 PMC9562569

[5] Gill T, Pollard AJ, Baker J, Tredwin C. Cracked tooth syndrome: assessment, prognosis and predictable management strategies. Eur J Prosthodont Restor Dent. 2021;29(4):209–17. 10.1922/EJPRD_2232Gill09 33770422

[6] Tian X, Qiao J, Guo N, Liu K, Li K. CBCT imaging and root canal treatment for taurodontism in mandibular second molar – a case report and literature review. J Radiol Case Rep. 2023;17(11):1–7. 10.3941/jrcr.v17i11.5212 PMC11022752 38638554

[7] Siqueira JF, Jr, Silva WO, Romeiro K, Gominho LF, Alves FRF, Rôças IN. Apical root canal microbiome associated with primary and posttreatment apical periodontitis: a systematic review. Int Endod J. 2024;57(8):1043–58. 10.1111/iej.14071 38634795

[8] Essam O, Boyle EL, Whitworth JM, Jarad FD. The Endodontic Complexity Assessment Tool (E-CAT): a digital form for assessing root canal treatment case difficulty. Int Endod J. 2021;54(7):1189–99. 10.1111/iej.13506 33682086

[9] Arul B, Suresh N, Sivarajan R, Natanasabapathy V. Influence of volume of endodontic irrigants used in different irrigation techniques on root canal dentin microhardness. Indian J Dent Res. 2021;32(2):230–5. 10.4103/ijdr.IJDR_709_18 34810395

[10] Van Nieuwenhuysen JP, D’Hoore W, Leprince JG. What ultimately matters in root canal treatment success and tooth preservation: a 25-year cohort study. Int Endod J. 2023;56(5):544–57. 10.1111/iej.13895 36683563

[11] Shao L, Yu Y. Development of a prediction nomogram model of recurrent febrile seizures in pediatric children. Eur J Pediatr. 2023;182(11):4875–88. 10.1007/s00431-023-05133-7 37597045

[12] Ye L, Xu X, Liu L, Chen F, Xia G. A nomogram for predicting cancer-related cognitive impairment in lung cancer patients from a nursing science precision health model perspective. Support Care Cancer. 2025;33(4):320. 10.1007/s00520-025-09383-z 40133674

[13] Zhuo J, Zhang W, Xu Y, Zhang J, Sun J, Ji M, et al. Nomogram for predicting post-traumatic hydrocephalus after decompressive craniectomy for traumatic brain injury. Rev Assoc Med Bras (1992). 2022;68(1):37–43. 10.1590/1806-9282.20210392 35239935

[14] Duncan HF, Nagendrababu V, El-Karim I, Dummer PMH. Outcome measures to assess the effectiveness of endodontic treatment for pulpitis and apical periodontitis for use in the development of European Society of Endodontology S3-level clinical practice guidelines: a consensus-based development. Int Endod J. 2021;54(12):2184–94. 10.1111/iej.13627 34553383

[15] Åström M, Thet Lwin ZM, Teni FS, Burström K, Berg J. Use of the visual analogue scale for health state valuation: a scoping review. Qual Life Res. 2023;32(10):2719–29. 10.1007/s11136-023-03411-3 37029258 PMC10474194

[16] Asgary S, Eghbal MJ, Shahravan A, Saberi E, Baghban AA, Parhizkar A. Outcomes of root canal therapy or full pulpotomy using two endodontic biomaterials in mature permanent teeth: a randomized controlled trial. Clin Oral Investig. 2022;26(3):3287–97. 10.1007/s00784-021-04310-y PMC8636579 34854987

[17] Ricucci D, Rôças IN, Alves FRF, Cabello PH, Siqueira JF, Jr. Outcome of direct pulp capping using calcium hydroxide: a long-term retrospective study. J Endod. 2023;49(1):45–54. 10.1016/j.joen.2022.11.005 36375647

[18] López-Valverde I, Vignoletti F, Vignoletti G, Martin C, Sanz M. Long-term tooth survival and success following primary root canal treatment: a 5- to 37-year retrospective observation. Clin Oral Investig. 2023;27(6):3233–44. 10.1007/s00784-023-04938-y PMC10264502 36933044

[19] Sim IG, Lim TS, Krishnaswamy G, Chen NN. Decision making for retention of endodontically treated posterior cracked teeth: a 5-year follow-up study. J Endod. 2016;42(2):225–9. 10.1016/j.joen.2015.11.011 26723485

[20] Bryce ÚM, Quinn BM, Edwards DC. Does single-visit root canal treatment of permanent teeth provide more benefit than a multiple-visit approach? Evid Based Dent. 2023;24(2):71–2. 10.1038/s41432-023-00888-2 37188920

[21] Chen YT, Hsu TY, Liu H, Chogle S. Factors related to the outcomes of cracked teeth after endodontic treatment. J Endod. 2021;47(2):215–20. 10.1016/j.joen.2020.11.024 33275995

[22] Seet RF, Chan PY, Khoo ST, Yu VSH, Lui JN. Characteristics of cracked teeth with reversible pulpitis after orthodontic banding – a prospective cohort study. J Endod. 2022;48(12):1476–85. 10.1016/j.joen.2022.09.002 36150561

[23] Rathke A, Frehse H, Hrusa B. Vertical root fracture resistance and crack formation of root canal-treated teeth restored with different post-luting systems. Odontology. 2022;110(4):719–25. 10.1007/s10266-022-00709-5 35523910 PMC9463252

[24] Krell KV, Caplan DJ. 12-month success of cracked teeth treated with orthograde root canal treatment. J Endod. 2018;44(4):543–8. 10.1016/j.joen.2017.12.025 29429822

[25] Lee TY, Yang SE, Kim HM, Kye MJ. Characteristics, treatment, and prognosis of cracked teeth: a comparison with data from 10 years ago. Eur J Dent. 2021;15(4):694–701. 10.1055/s-0041-1728842 34171933 PMC8630975

[26] Efthimiou O, Seo M, Chalkou K, Debray T, Egger M, Salanti G. Developing clinical prediction models: a step-by-step guide. BMJ. 2024;386:e078276. 10.1136/bmj-2023-078276 39227063 PMC11369751

